# Association of Impaired Reactive Aldehyde Metabolism with Delayed Graft Function in Human Kidney Transplantation

**DOI:** 10.1155/2018/3704129

**Published:** 2018-12-23

**Authors:** Leonie G. M. Wijermars, Alexander F. Schaapherder, Thomas George, Pritam Sinharoy, Eric R. Gross

**Affiliations:** ^1^Department of Surgery, Leiden University Medical Center, PO Box 9600, 2300 RC Leiden, Netherlands; ^2^Department of Anesthesiology, Perioperative and Pain Medicine, School of Medicine, Stanford University, 300 Pasteur Drive H3580, Stanford, 94305-5640 California, USA

## Abstract

Delayed graft function is an early complication following kidney transplantation with an unclear molecular mechanism. Here we determined whether impaired reactive aldehyde metabolism is associated with delayed graft function. Human kidney biopsies from grafts with delayed graft function were compared with grafts that did not develop delayed graft function by Ingenuity gene pathway analysis. A second series of grafts with delayed graft function (*n* = 10) were compared to grafts that did not develop delayed graft function (*n* = 10) by measuring reactive aldehyde metabolism, reactive aldehyde-induced protein adduct formation, and aldehyde dehydrogenase (ALDH) gene and protein expression. In the first series of kidney biopsies, several gene families known for metabolizing reactive aldehydes, such as aldehyde dehydrogenase (ALDH), aldo-keto reductase (AKR), and glutathione-S transferase (GSTA), were upregulated in kidneys that did not develop delayed graft function versus those that did. In the second series of kidney grafts, we focused on measuring aldehyde-induced protein adducts and ALDH enzymatic activity. The reactive aldehyde metabolism by ALDH enzymes was reduced in kidneys with delayed graft function compared to those that did not (37 ± 12^∗^ vs. 79 ± 5 *μ*g/min/mg tissue, ^∗^ *P* < 0.005, respectively). ALDH enzymatic activity was also negatively correlated with length of hospital stay after a kidney transplant. Together, our study identifies a reduced ALDH enzymatic activity with kidneys developing delayed graft function compared to those that did not. Measuring ALDH enzymatic activity and reactive aldehyde-induced protein adducts can potentially be further developed as a biomarker to assess for delayed graft function and recovery from a kidney transplant.

## 1. Introduction

Organ transplantation, including kidney transplantation, is a known and planned occurrence of organ reperfusion injury. Reperfusion injury in turn can trigger delayed graft function, resulting in the deferred functional recovery of the donor kidney following kidney transplantation.

Delayed graft function detrimentally affects renal function and graft longevity and is a risk factor for acute kidney rejection [1, 2]. The incidence of delayed graft function in deceased donor kidneys is ~25% and perhaps as high as 50% for kidneys from cardiac death donor organs [3, 4]. Additionally, the incidence of delayed graft function is steadily rising due to the increased use of marginal donor grafts secondary to organ transplant shortages. As a consequence, delayed graft function leads to reduced graft function, prolonged hospital admissions, increased demand of donor kidneys for retransplantation secondary to rejection, and a higher economic societal burden [5]. Several definitions exist for delayed graft function [6]. For this manuscript, we define delayed graft function as the requirement of dialysis within the first 7 postoperative days of a kidney transplant [6].

During reperfusion of an organ, reactive oxygen species produced at the mitochondria causes lipid peroxidation which generates reactive aldehydes which can impair cellular functions [7–9]. The reactive aldehydes produced include acetaldehyde, malondialdehyde (MDA), and 4-hydroxynonenal (4-HNE). To metabolize these reactive aldehydes, several protein families such as glutathione-S reductase, aldo-keto reductase, and aldehyde dehydrogenase gene families contribute to metabolizing aldehydes produced within the cell [7, 10, 11].

Although kidney transplants from living donors are less susceptible to delayed graft function when compared to grafts from deceased donors [1], the differences in molecular biology have not been extensively studied. Here we used human renal biopsies from transplanted deceased donor kidneys who developed delayed graft function and compared the molecular differences using gene arrays to kidneys from living donor transplants that did not develop delayed graft function. From this approach, we here suggest that impaired reactive aldehyde metabolism by ALDH enzymes is associated with delayed graft function.

## 2. Materials and Methods

### 2.1. Patient Enrollment

The study protocol was approved by the medical ethics committee at the Leiden University Medical Center. Written informed consent was obtained from each patient.

**Figure 1:**
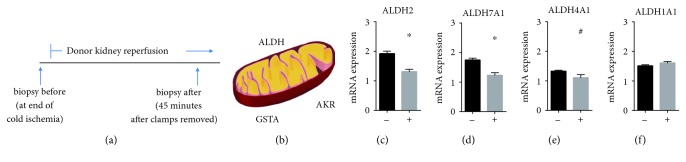
Changes in ALDH enzymes found by gene array studies. (a) Experimental scheme for biopsies. 10 paired renal biopsies from kidneys that did not develop delayed graft function and 8 paired renal biopsies that developed delayed graft function were obtained. One biopsy was taken prior to transplant at the end of cold ischemia. The second biopsy was taken after transplant and reperfusion 45 minutes after the cross-clamp was removed. (b) Of the genes identified by Ingenuity pathway analysis, several gene families are identified to be involved in reactive aldehyde metabolism. (c), (d) ALDH2 and ALDH7A1 were statistically significant between kidneys that did not develop delayed graft function compared to kidneys developing delayed graft function (^∗^ *P* < 0.01). (e) ALDH4A1 nearly reached statistical significance (^#^ *P* = 0.05). (f) ALDH1A1, another ALDH family member, did not change between living donor kidneys compared to kidneys that developed delayed graft function. (-) = donor kidneys without delayed graft function; (+) = donor kidneys with delayed graft function.

Paired renal cortical biopsies were obtained at the end of the cold ischemic period (prior to implantation) and 45 min after reperfusion of the kidney in the recipient. Kidney biopsies from an initial 18 donor kidneys were used to conduct the whole genome array portion of the study (Figure 1(a)). We then obtained an additional 10 paired renal biopsies from living donor kidneys that did not develop delayed graft function and 10 paired renal biopsies from deceased donor kidneys that developed delayed graft function. Details regarding enrollment and patient demographics are described in detail (Tables 1 and 2).

Renal allografts were perfused and stored with either University of Wisconsin solution or Custodiol® HTK (histidine–tryptophan–ketoglutarate) solution. None of the grafts were machine perfused. For renal transplantation, all patients were induced by propofol, sufentanil, and atracurium. Patients were intubated in addition to a central venous catheter used for intraoperative monitoring. Patients received basiliximab (day 0 and 4) as immunosuppressive induction. Patients were maintained on tacrolimus or cyclosporine A, mycophenolate mofetil, and steroids for immunosuppression.

Biopsies were taken from the upper pole of the kidney. For biopsies taken after reperfusion, a spring-loaded automatic biopsy needle was used (16 Ga Travenol). Tissue was snap frozen in liquid nitrogen and stored at −80°C and labeled with a unique identifier, blinding the person performing the analysis. For the genome array studies, all samples were analyzed together after the groups were collected. The validation studies were also performed after the tissue was obtained, selectively using only tissue from deceased donors who developed delayed graft function after transplantation.

The transplanted patients were also followed during their hospital stay. The length of hospital stay after transplantation in addition to glomerular filtration rate at postoperative day 7 was also assessed. Patients were considered as developing delayed graft function if they were in need of dialysis within the first week after transplantation. For those requiring dialysis, acute rejection was excluded as a cause of delayed graft function by renal biopsy. Surgical complications of the transplant procedure were also excluded as a source of delayed graft function. Further, if transplant recipients required one episode of dialysis after transplantation due to incident hyperkalemia, these grafts were also not included as having delayed graft function.

### 2.2. Genome Array

Paired biopsies were taken for the patients undergoing renal transplantation with one biopsy prior to transplantation and one biopsy 45 minutes after transplant reperfusion. From these renal biopsies, total RNA was extracted using RNAzol (Campro Scientific, Veenendaal, Netherlands) and glass beads. The integrity of each RNA sample was examined by Agilent Lab-on-a-chip technology using the RNA 6000 Nano LabChip kit and a Bioanalyzer 2100 (Agilent Technologies, Amstelveen, Netherlands). RNA preparations were considered suitable for array hybridization only if samples showed intact 18S and 28S rRNA bands and displayed no chromosomal peaks or RNA degradation products (RNA integrity number >8.0). Microarray analysis was performed using Illumina whole-genome gene expression BeadChips (Illumina BeadArray®, San Diego, USA) according to the manufacturer's instructions at the Service XS facility in Leiden.

The tissue biopsies collected from the two groups were run on separate gene arrays. The 2 paired tissue biopsies obtained for each transplanted kidney resulted in running 36 gene arrays for the tissue biopsies that were collected. The gene arrays obtained were analyzed by a statistician at the University of Leiden blinded to the identification of the groups of tissue biopsies collected. An average replicate value was calculated and log2 ratios were computed by comparing the matched pair biopsies and the gene expression differences for each transplanted kidney prior to and after reperfusion. One value per gene was calculated for the average expression of multiple probes with the same Entrez gene identification, resulting in 15,093 unique gene profiles. The unique gene profiles obtained from the whole genome array were analyzed by Ingenuity pathway analysis (Redwood City, CA, USA).

### 2.3. Biochemical Assays

In addition to biopsies collected for gene array studies, 20 transplanted kidneys were used to perform further biochemical analysis which included qPCR, western blot (for aldehyde-induced protein adduct and protein expression), and aldehyde enzymatic activity assays.

For qPCR, RNA was isolated from kidney biopsies taken at the end of the cold ischemic period. Kidney biopsy lysates were made by sonification, and RNA was isolated by the use of Ambion© RNAqueous Kit. To substantially reduce the possibility of DNA contamination in the preparations, the isolated total RNA was subject to precipitation with lithium chloride and DNase digestion (Ambion DNase-free). cDNA was made using the Takara© Primescript cDNA synthesis kit with oligo dT primers. qPCR reactions were performed in a final volume of 20 *μ*l that contained 15 *μ*l of Fast SYBR© Green Master Mix (Life Technologies), 1000 nM primer (ALDH2, ALDH7A1, or GAPDH) or 500 nM primer (ALDH4A1), and 10 ng cDNA. The cycling protocol was 20 seconds at 95°C, followed by 40 cycles of 3 seconds at 95°C and 30 seconds at 61°C. The melt curve protocol was 15 seconds at 95°C, followed by a minute at 60°C, followed by a gradual temperature increase from 60°C to 95°C (+0.03°C per 15 seconds) in 42 minutes.

Western blot analysis was used to quantify 4-HNE-induced protein adducts in addition to protein expression of ALDH enzymes. For western blot analysis, kidney biopsies taken 45 minutes after reperfusion were homogenized in mannitol-sucrose buffer (210 mM mannitol, 70 mM sucrose, MOPS 5 mM, and EDTA 1 mM, pH 7.4) with protease and phosphatase inhibitors (1 : 300, Sigma protease inhibitor cocktail, Sigma phosphatase inhibitor cocktail 2, and phosphatase inhibitor cocktail 3) using a glass homogenizer (Wheaton, 1 mL tissue grinder). Homogenates were subsequently collected and spun at 800 g × 10 minutes to remove nuclei and debris. The supernatant was collected and protein counts were obtained by Bradford assay and samples were normalized to *μ*g protein. Western blot was performed as described [12]. Primary antibodies used included anti-4HNE (rabbit, Alpha Diagnostics, 1 : 500), ALDH2 (goat, Santa Cruz, 1 : 1000), ALDH4A1 (rabbit, Abcam #EPR14287, 1 : 1000), ALDH7A1 (rabbit, Abcam, #EP1934Y, 1 : 1000), and ALDH1A1 (rabbit, Abcam, #EP1993Y, 1 : 1000). Secondary antibodies were used at 1 : 3000 consisting of anti-goat (Santa Cruz) and anti-rabbit (Sigma). Density of bands was measured by ImageJ and normalized to GAPDH (Santa Cruz, #47724, 1 : 3000).

To determine ALDH enzyme activity, 25 *μ*g of protein were used. ALDH enzyme activity was measured spectrophotometrically (340 nm) by analyzing the reaction of NAD+ to NADH as previously described [7, 12]. The activity assay was performed at 25°C in 50 mM sodium pyrophosphate buffer (pH 9.4), 2.5 mM NAD+, and 10 mM acetaldehyde was used as substrate. ALDH enzyme activity was converted to *μ*mole NADH/min/mg of protein.

### 2.4. Statistical Analysis

Sample sizes were chosen for this study based on previous experience the University of Leiden has on conducting clinical studies regarding ischemia-reperfusion injury in human kidney transplantation [13, 14]. SPSS 22.0 (SPSS, Chicago, IL) was used for gene array statistical analysis. For pathway analysis, data is represented as *P* values for the change in expression comparing the paired biopsies, fitting into predefined pathways. *P* values are expressed as the –log *P* value. Results were then analyzed by biostatistical methods using average replicate values for each group of samples. Log2 ratios were computed and one value per gene was calculated for the average expression of probes with the same Entrez gene ID. For qPCR and protein assays, data was analyzed with GraphPad prism.

## 3. Results

Changes in gene pathway *p*-values using Ingenuity pathway analysis were calculated and initially reported [15]. Here, we analyzed the gene array data by comparing the gene array of a biopsy taken 45 minutes after reperfusion compared to the paired biopsy gene array taken at the end of cold ischemia. When looking at these differences for each group, we identified 10 signaling pathways that were induced with the highest differences in *p*-values in kidneys which did not develop delayed graft function compared to the kidneys which developed delayed graft function (Table 3). In particular, when examining these differences, distinct genes were upregulated for only the kidney biopsies that did not develop delayed graft function at reperfusion when compared to the kidney biopsies that developed delayed graft function for these pathways (Table 3).

Genes that contribute to aldehyde metabolism were identified in this gene array including the family of aldehyde dehydrogenase enzymes (ALDH2, ALDH4A1, and ALDH7A1), aldo-keto reductases (AKR1A1), and glutathione-S-transferases (GSTA1, GSTA2, GSTA3, and GST5) known to metabolize reactive aldehydes typically generated by the mitochondria during reperfusion (Figure 1(b)). Together, these results suggest that differences exist in the reactive aldehyde production and metabolism within donor kidneys that developed delayed graft function versus those that did not.

We further examined the gene array expression for ALDH2, ALDH4A1, and ALDH7A1 in addition to the other ALDH family of enzymes. Other ALDH enzymes (besides the cytosolic enzymes ALDH3A2 and ALDH1L1 unknown to be involved in aldehyde metabolism) remained unchanged (Supplemental Figure 1). ALDH2 and ALDH7A1 were significantly higher in the living donor grafts compared to the grafts with delayed graft function (ALDH2: 1919.4 ± 86.7 versus 1314.5 ± 76.6^∗^, ALDH7A1: 1739.9 ± 65.5 versus 1230.4 ± 83.1^∗^, ^∗^ *P* = 0.001) with ALDH4A1 just at statistical significance (1324.1 ± 34.7 versus 1102.9 ± 105.9; *p* = 0.05, Figures 1(c)–1(e)). As a comparison, no differences were seen for the cytosolic ALDH enzyme ALDH1A1 (Figure 1(f)).

**Figure 2:**
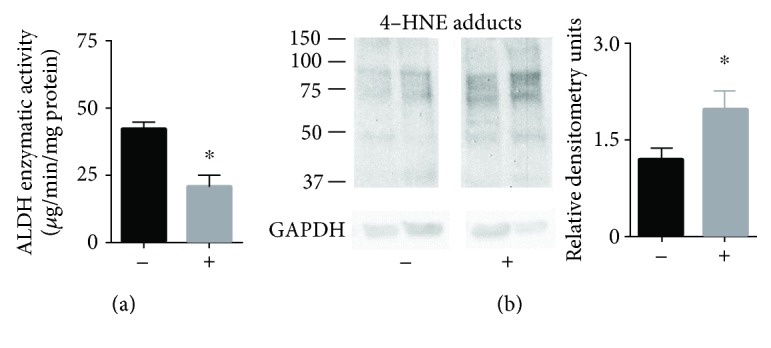
ALDH enzyme activity and 4-HNE protein-induced adduct formation taken from biopsies of kidneys 45 minutes after reperfusion. (a) ALDH enzymatic activity when challenged with acetaldehyde. *n* = 8/group, ^∗^ *P* < 0.005; (b) 4-hydroxynonenal-induced protein adducts. Representative western blot in addition to quantification by densitometry. Western blots were normalized to GAPDH. (-) = donor kidneys without delayed graft function; (+) = donor kidneys with delayed graft function, *n* = 10/group, ^∗^ *P* < 0.05.

Since the gene array findings suggest a link to reactive aldehyde metabolism, we further examined reactive aldehyde metabolism and aldehyde-induced protein adducts by obtaining paired biopsies from additional grafts that did not develop delayed graft function and donor kidney grafts that developed delayed graft function. For these biopsies, we determined the ALDH-dependent enzymatic activity to metabolize reactive aldehydes (using the substrate NAD+) in addition to 4-hydroxynonenal- (4-HNE-) induced protein adducts for biopsies taken 45 minutes after reperfusion. The total ALDH enzymatic activity was significantly higher for donor kidney biopsies without delayed graft function when compared to biopsies from kidneys that developed delayed graft function (Figure 2(a): 78.6 ± 4.7 vs. 36.9 ± 11.5^∗^ *μ*g/min/mg protein, *n* = 8/group, ^∗^ *P* < 0.005). Additionally, the amount of 4-hydroxynonenal-induced protein adducts in the reperfused kidneys was significantly higher for kidneys developing delayed graft function versus those that did not (Figure 2(b), 4-HNE adducts: 1.2 ± 0.2 vs. 1.9 ± 0.3^∗^, *n* = 10/group, ^∗^ *P* < 0.028, *n* = 10/group).

Since a full analysis of the ALDH, AKR, and GSTA enzyme classes identified through gene array was not feasible to examine in human tissue biopsies based upon the biopsy sample size, we chose 3 candidate genes from our array that appeared in high frequency within the signaling pathways. These members of the aldehyde dehydrogenase gene family, ALDH2, ALDH7A1, and ALDH4A1, metabolize reactive aldehydes in a NAD-dependent fashion and can protect against cellular stress [7, 8, 16, 17]. Therefore, we focused on examining expression for the ALDH enzymes ALDH2, ALDH4A1, and ALDH7A1 with ALDH1A1 used as a control.

**Figure 3:**
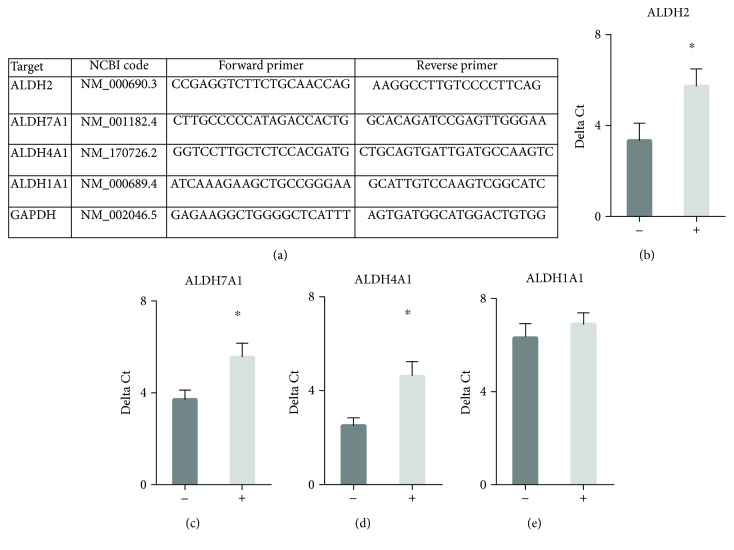
qPCR studies for kidney biopsies taken prior to transplant. For a separate series of kidney transplants, biopsies were collected for qPCR. (a) PCR primers used for qPCR. qPCR reactions were performed in a final volume of 20 *μ*l that contained 15 *μ*l of Fast SYBR© Green Master Mix (Life Technologies), 1000 nM primer (ALDH2, ALDH7A1 or GAPDH) or 500 nM primer (ALDH4A1), and 10 ng cDNA. (b–e) qPCR results for (b) ALDH2, (c) ALDH7A1, (d) ALDH4A1, and (e) ALDH1A1. ALDH2, ALDH7A1, and ALDH4A1 by qPCR were significantly elevated in kidneys that did not develop delayed graft function versus those kidneys that developed delayed graft function (*n* = 10/group, ^∗^ *P* < 0.05). Ct values of each gene were normalized to GAPDH to calculate the delta Ct value. (-) = donor kidneys without delayed graft function; (+) = donor kidneys with delayed graft function.

We performed qPCR on all biopsy samples obtained prior to kidney transplantation. The details of the primer design and validation for ALDH2, ALDH4A1, ALDH7A1, and ALDH1A1 are described (Figure 3(a) and Supplemental Figure 2). Significant differences were noted when calculating a delta Ct (normalized to GAPDH) for donor kidneys that did not develop delayed graft function when compared to those donor kidneys that developed delayed graft function (Figures 3(b)–3(d), *n* = 10/group, ALDH2: 3.3 ± 0.8 vs. 5.7 ± 0.8^∗^, ALDH7A1 3.7 ± 0.4 vs. 5.6 ± 0.6^∗^, and ALDH4A1: 2.5 ± 0.3 vs. 4.6 ± 0.6^∗∗^, ^∗^ *P* < 0.01, ^∗∗^ *P* < 0.001, reported as delta Ct values normalized to GAPDH). As a comparison, ALDH1A1 was unchanged (Figure 3(e)).

**Figure 4:**
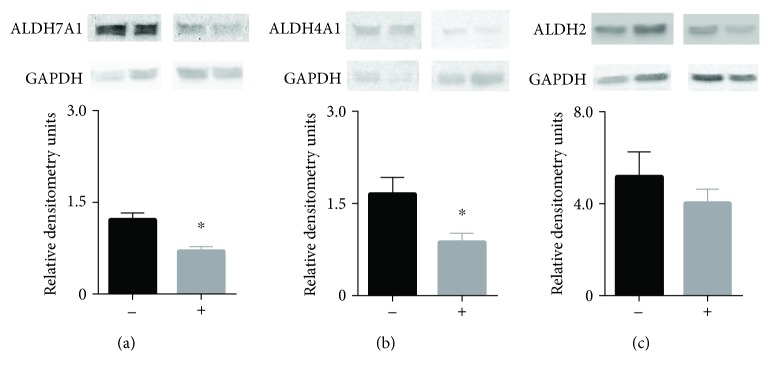
Western blot of ALDH7A1, ALDH4A1, and ALDH2 taken from biopsies of kidneys 45 minutes after reperfusion. (a–c) Western blots for (a) ALDH7A1, (b) ALDH4A1, and (c) ALDH2. All western blots were normalized to GAPDH. Both ALDH7A1 and ALDH4A1 were significantly different in kidneys that did not develop delayed graft function versus those kidneys that developed delayed graft function. (-) = donor kidneys without delayed graft function; (+) = donor kidneys with delayed graft function, *n* = 10/group, ^∗^ *P* < 0.05.

We also quantified the levels of protein expression by western blot for ALDH2, ALDH7A1, and ALDH4A1. Both ALDH7A1 and ALDH4A1 had significant changes in protein expression between the two groups (Figure 4(a), ALDH7A1: 1.2 ± 0.1^∗^ vs. 0.7 ± 0.07, ^∗^ *P* < 0.001, Figure 4(b), ALDH4A1: 1.7 ± 0.3^∗^ vs. 0.9 ± 0.1, ^∗^ *P* < 0.017). Western blot for ALDH2 also showed a relative change in expression that did not reach statistical significance (Figure 4(c), ALDH2: 5.2 ± 1.1 vs. 4.0 ± 0.6).

**Figure 5:**
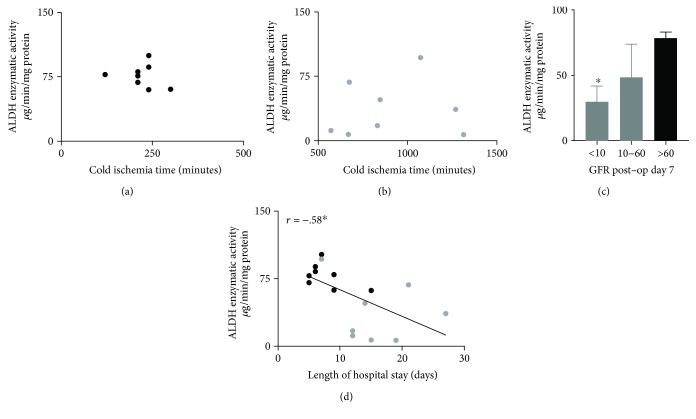
Association of ALDH enzymatic activity with transplant outcomes. (a) The level of ALDH enzymatic activity measured from the renal biopsy at 45 minutes after reperfusion during transplantation was associated with GFR at day 7 (GFR <10 *n* = 5, GFR 10-6 *n* = 3, GFR >60 *n* = 8. ^∗^ *P* = 0.013 versus >60 GFR group). (b) ALDH enzymatic activity was also negatively correlated with the length of hospital stay. *n* = 16, ^∗^ *P* = 0.0183.

Since our results suggested that impaired reactive aldehyde metabolism may be associated with delayed graft function, we further examined whether cold ischemia times influenced ALDH enzymatic activity. Generally, the cold ischemia times in the kidneys that did not develop delayed graft function was less compared to the kidneys that developed delayed graft function (Table 2, living donor without delayed graft function: 221 ± 18 minutes versus deceased donor with delayed graft function: 900 ± 88^∗^ minutes, ^∗^ *P* < 0.01). No correlation between ALDH enzymatic activity and cold ischemia times for living donor grafts without delayed graft function was found, and the range of cold ischemia time for these kidneys was small (Figure 5(a) 6 of 8 samples had cold ischemia times between 210-240 minutes). Further, in grafts that did develop delayed graft function, a wider range of cold ischemia times existed; however, no association between ALDH enzymatic activity and cold ischemia times was found (Figure 5(b)).

However, ALDH enzymatic activity was also examined in relation to glomerular filtration rate (GFR) measured in patients at postoperative day 7 and length of hospital stay. Interestingly, those kidney biopsies that had lower ALDH enzymatic activity also had a lower GFR at day 7 (Figure 5(c): GFR <10: 30 ± 12^∗^, GFR 10-60: 49 ± 25, GFR >60: 79 ± 5 *μ*g/min/mg protein, *n* = 5, *n* = 3, and *n* = 8, respectively, ^∗^ *P* = 0.013 vs. GFR >60 group). In addition, an inverse association also existed with ALDH enzymatic activity when compared to the length of hospital stay (Figure 5(d): *r* = −0.58 by Pearson coefficient, *P* = 0.0183).

## 4. Discussion

Here we describe an important role by enzymes within the human kidney to metabolize reactive aldehydes produced during reperfusion of an organ transplant. We suggest quantifying the enzymatic activity to metabolize reactive aldehydes coupled with reactive aldehyde-induced protein adduct levels may be useful in possibly predicting delayed graft function and recovery from a kidney transplant. Our results suggest that measuring the overall reactive aldehyde balance within the donor kidney may be important in understanding which transplants may be at risk for developing delayed graft function. These initial findings may potentially lead to developing a cellular biomarker based on reactive aldehyde production and metabolism to predict delayed graft function.

Presently, several biomarkers are being investigated for their ability to predict delayed graft function including neutrophil gelatinase-associated lipocalin (NGAL), kidney injury molecule 1 (KIM-1), interleukin18 (IL-18), klotho, cystatin C, and liver type fatty acid binding protein (L-FABP) [18–20]. Recently, NGAL blood levels taken from brain-dead kidney donors prior to kidney graft harvesting could not predict the development of delayed graft function [20]. Additionally, the idea of combining several biomarkers to detect delayed graft function was proposed, with a possible triple biomarker approach of serum malondialdehyde, cystatin C, and creatinine [21].

Although associations exist between serum malondialdehyde levels and delayed graft function [21, 22], serum malondialdehyde may underestimate the cellular damage contributed to lipid peroxidation. This is since ~95% of malondialdehyde is in the bound form with only 5% being free to measure in the serum for patients with end-stage renal disease [23]. Therefore, assessing both reactive aldehyde-induced protein levels and reactive aldehyde metabolism may provide a means to develop complementary biomarkers in order to predict delayed graft function. Even though our results were from kidney biopsies and not measured from circulating blood levels, we suggest that measuring the state of reactive aldehyde production and metabolism may potentially be useful in predicting delayed graft function rather than assessing specific protein biomarkers or reactive aldehyde levels within the blood.

Our data also examined ALDH7A1, ALDH4A1, and ALDH2 within the ALDH enzyme family suggesting that these enzymes are involved and contribute to a decreased reactive aldehyde metabolism seen in kidneys developing delayed graft function. However, the contribution of other families of enzymes such as AKR and GSTA cannot be excluded. Although protein expression was not significantly changed for ALDH2 in our study, it cannot be ruled out that ALDH2 was posttranslationally modified by reactive aldehydes. To identify posttranslational modifications, immunoprecipitation of an enzyme (such as ALDH2) followed by western blot for aldehyde-induced protein adducts could be performed. However, the amount of protein needed to conduct this type of study exceeded the amount obtained from a renal biopsy for our study. Based upon prior studies performed *in vitro*, 4-HNE can inhibit human recombinant ALDH2 resulting in a decreased capability for the enzyme to metabolize reactive aldehydes [24]. At 50 *μ*M 4-HNE, most of the effect is reversible; however, at 500 *μ*M 4-HNE concentrations, this effect is noted to be irreversible [24]. Since with increases in oxidative stress blood levels of 4-HNE in humans can increase 10- to 100-fold [25], the reactive aldehydes generated during kidney reperfusion likely can cause a partial inhibition of ALDH enzymes. This inhibition can potentially contribute to the decreased reactive aldehyde metabolism we identified for our study in kidneys that developed delayed graft function.

In the era of precision medicine, it is also important to determine how genetic polymorphisms in mitochondrial ALDH enzymes may affect reactive aldehyde metabolism and delay graft function. In particular, ~560 million people in the world have a genetic variant of ALDH2, ALDH2^∗^2, which severely limits the metabolism of reactive aldehydes [26]. Although no study has associated with an ALDH2^∗^2 variant as a predictor of delayed graft function, this may be due to organ transplantation (and in particular kidney transplantation) numbers that are traditionally low in East Asia compared to the rest of the world secondary to cultural reasons [27]. Further, very little is known regarding whether a genetic polymorphism in ALDH7A1 which decreases the enzymatic activity may also affect cellular function during organ transplantation. However, ALDH7A1 overexpression protects from both cellular toxicity and hyperosmotic stress [16, 28]. Therefore, the effects of either the donor or recipient having a genetic polymorphism in mitochondrial ALDH enzymes warrants further study [7, 12].

Our study does have a number of potential limitations that need to be considered when interpreting the data presented. Although we show an importance of reactive aldehyde production and metabolism in delayed graft function, we could not conclusively identify all the enzymes involved based on the size of the kidney biopsy obtained. Therefore, we only focused on 3 ALDH enzymes even though AKR and GSTA family members also had an increased gene expression for kidneys that did not develop delayed graft function. Additionally, the decreased protein expression observed might also be due to loss of cell and mitochondrial integrity since donor death itself induces metabolic dysregulation and mitochondrial dysfunction. We also compared grafts from living donor kidneys to deceased donor kidneys that developed delayed graft function. Further studies will be needed to compare reactive aldehyde production and metabolism also in deceased donor kidneys that did not develop delayed graft function as the effect we identify may be secondary to comparing living donor kidneys to deceased donor kidneys.

The study is also an association study and will require further validation both in experimental models and in the clinical realm. Although we did find associations for aldehyde enzymatic activity with GFR and length of hospital stay, these associations should be considered with caution as many factors contribute to the postoperative recovery from a kidney transplant and a larger study would be important to confirm these findings.

**Figure 6:**
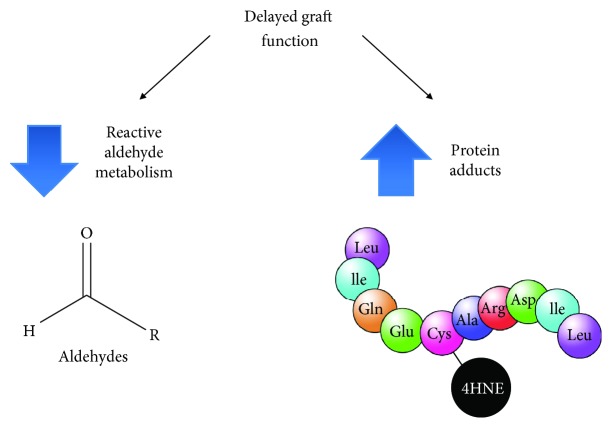
Summary. Reactive aldehyde metabolism is important in clearing toxic reactive aldehydes including 4-HNE in transplanted kidneys. Impaired reactive aldehyde metabolism and an increase in 4-HNE-induced protein adducts were found to be associated with delayed graft function.

Together, we suggest that a decreased ability to metabolize reactive aldehydes in donor kidneys that develop delayed graft function occurs (Figure 6). This diminished metabolism of reactive aldehydes can result in aldehyde adducts forming on proteins that can potentially impair cellular functions by producing changes in enzyme activity, ion channel gating, and mitochondrial energetics [7, 29–31].

## Figures and Tables

**Table 1:** Patient characteristics for gene array studies.

	Living donor kidney transplant (*n* = 10)	Deceased donor kidney transplant (*n* = 8)
Age of recipient (yrs)	49 ± 5	56 ± 4
Sex of recipient (% males)	60%	50%
Age of donor (yrs)	52 ± 2	49 ± 6
Sex of donor (% males)	30%	75%
Cold ischemia time (min)	213 ± 12	1001 ± 96^∗^
Hospital stay (days)	8 ± 1	15 ± 2^∗^
*Recipient cause of renal failure*		
Glomerulonephritis	40%	12.5%
Polycystic kidney disease	20%	25%
Diabetes mellitus type 2	10%	12.5%
Obstructive uropathy	10%	0%
Malignant hypertension	0%	0%
Renal failure	20%	50%
*Donor cause of death*		
Living donor	100%	
CVA		0%
SAB		25%
Trauma		37.5%
CA-OHCA-AMI		25%
Suicide		0%
Miscellaneous		12.5%
*Histocompatibility (HLA mismatches, %)*		
0	10%	12.5%
1	10%	25%
2	10%	25%
3	20%	25%
4	20%	12.5%
5	20%	0%
6	10%	0%

A total of 18 patients were included. When comparing recipients of a living donor transplant to recipients of a deceased donor transplant, significant differences were noted for duration of ischemia time, and length of posttransplantation hospital stay. ^∗^ *P* < 0.01, CVA = cerebral vascular accident, SAB = subarachnoid bleeding, CA = cardiac arrest, OHCA = out of hospital cardiac arrest, AMI = acute myocardial infarction. Data represents mean ± SEM.

**Table 2:** Patient characteristics for validation studies.

	Living donor kidney transplant (*n* = 10)	Deceased donor kidney transplant (*n* = 10)
Age of recipient (yrs)	58 ± 4	56 ± 4
Sex of recipient (% males)	70%	60%
Age of donor (yrs)	58 ± 2	57 ± 4
Sex of donor (% males)	50%	70%
Cold ischemia time (min)	221 ± 18	900 ± 88^∗^
Hospital stay (days)	8 ± 2	15 ± 3^∗^
*Recipient cause of renal failure*		
Glomerulonephritis	40%	30%
Polycystic kidney disease	20%	20%
Diabetes mellitus type 2	0%	20%
Obstructive uropathy	10%	10%
Malignant hypertension	10%	10%
Renal failure	20%	10%
*Donor cause of death*		
Living donor	100%	
CVA		20%
SAB		20%
Trauma		20%
CA-OHCA-AMI		30%
Suicide		10%
Miscellaneous		0%
*Histocompatibility (HLA mismatches, %)*		
0	0%	10%
1	10%	10%
2	0%	30%
3	10%	40%
4	20%	0%
5	40%	10%
6	20%	0%

A total of 20 patients were recruited. When comparing recipients of a living donor transplant to recipients of a deceased donor transplant, significant differences were noted for developing delayed graft function, duration of ischemia, and length of posttransplantation hospital stay. ^∗^ *P* < 0.01, CVA = cerebrovascular accident, SAB = subarachnoid bleeding, CA = cardiac arrest, OHCA = out of hospital cardiac arrest, AMI = acute myocardial infarction.

**Table 3:** Gene array differences from living compared to deceased donor kidney biopsies.

Pathway	Difference in value	Genes only upregulated in living donor	Genes only upregulated in deceased donor	Common
Serotonin degradation	6.59	ALDH4A1, ALDH2, ALDH7A1, ALDH3A2, ADH6, UGT2B7, UGT2B10, UGT2A3, UGT1A9, AKR1A1, SMOX, DHRS4	None	None

Tryptophan degradation	5.54	ALDH4A1, ALDH2, ALDH7A1, ALDH3A2, AKR1A1, DDC, SMOX	None	None

Histamine degradation	5.34	ALDH4A1, ALDH2, ALDH7A1, ALDH3A2, HNMT, ABP1	None	None

Ethanol degradation II	5.03	ALDH4A1, ALDH2, ALDH7A1, ALDH3A2, ADH6, AKR1A1, ACSS2, DHRS4	None	None

NRF-2-mediated oxidative stress response	4.82	AKR7A2, AKR1A1, FTL, NQO2, ABCC2, MAF, GSTA5, SCARB1, FMO1, GSTA1, GSTA2, GSTA3, MGST1, PRKCQ, ACTB, ACTG1, MGST2, MAP2K3, SQSTM1, AOX1, EIF2AK3, EPHX1	UBB, JUNB, DNAJA1, DNA	FOS, JUN, JUND, DNAJA4, DNAJB11, MAFF

Xenobiotic metabolism signaling	4.63	ALDH4A1, ALDH7A1, ALDH3A2, ALDH8A1, FTL, UGT2B7, NQO2, ABCC2, MAF, GSTA5, CYP3A7, HS6ST2, SMOX, FMO1, GSTA1, GSTA2, GSTA3, MGST1, PRKCQ, UGT2B10, UGT8, PPP2R5A, MGST2, MAP2K3, EIF2AK3, UGT1A9	CITED2, MAP3K8, TNF, HSP90AB1, HSP90AA1	None

Noradrenaline and adrenaline degradation	4.33	ALDH4A1, ALDH2, ALDH7A1, ALDH3A2, ALDH8A1, ADH6, AKR1A1, SMOX, DHRS4	None	None

LPS/IL-1-mediated inhibition of RXR function	4.14	ALDH4A1, ALDH7A1, ALDH3A2, ALDH8A1, GSTA1, GSTA2, GSTA3, GSTA5, APOE, MGST1, SLC27A2, ACOX2, ABCC2, CYP3A7, IL1R2, MGST2, SCARB1, NR5A2, HS6ST2, FMO1, SMOX	ALAS1, HMGCS1, TNF	ACSL3, JUN, NR0B2

Glutathoine-mediated detoxification	4.12	GSTA1, GSTA2, GSTA3, GSTA5, MGST1, MGST2, GGH	None	None

Oxidative ethanol degradation	3.98	ALDH4A1, ALDH2, ALDH7A1, ALDH3A2, ACSS2	None	ACSL3

Whole genome array changes before and after kidney transplantation were compared from each donor kidney that did not develop delayed graft function (living donor) with those that did develop delayed graft function (deceased donor) using Ingenuity pathway analysis. The difference in *P*-values between gene arrays sets were the highest for the 10 signaling pathways listed. Specific genes that were only upregulated in living donor kidneys and deceased donor kidneys are listed. Further, genes that were upregulated in both kidneys that developed delayed graft function versus those that did not are also listed.

## Data Availability

The data used to support the findings of this study are included within the article, supplemental information, and are cited at relevant places within the text as reference [13].

## Supplementary Materials

Supplementary Materials Microarray studies: microarray analysis was performed using Illumina whole-genome gene expression BeadChips. The gene array expression for the other ALDH family of enzymes on the microarray (besides ALDH2, ALDH4A1, and ALDH7A1 that were presented in the nonsupplemental portion of the manuscript) is presented in Supplemental Figure 1. qPCR primer validation: primer design and validation for ALDH2, ALDH4A1, ALDH7A1, and ALDH1A1 are presented in Supplemental Figure 2.
