# Supplementary material for: Association of Impaired Reactive Aldehyde Metabolism with Delayed Graft Function in Human Kidney Transplantation
Source: Oxid Med Cell Longev. 2018 Dec 23;2018:3704129. doi: 10.1155/2018/3704129 (PMC6323462; doi:10.1155/2018/3704129)
Supplement: Supplementary Materials — Microarray studies: microarray analysis was performed using Illumina whole-genome gene expression BeadChips. The gene array expression for the other ALDH family of enzymes on the microarray (besides ALDH2, ALDH4A1, and ALDH7A1 that were presented in the nonsupplemental portion of the manuscript) is presented in Supplemental Figure 1. qPCR primer validation: primer design and validation for ALDH2, ALDH4A1, ALDH7A1, and ALDH1A1 are presented in Supplemental Figure 2. [file 3704129.f1.pdf]

Supplemental Material:

Association of impaired reactive aldehyde metabolism with delayed graft function in human kidney transplantation

Leonie G.M. Wijermars, Bsc[1], Alexander F. Schaapherder, MD, PhD[1], Thomas George, BS[2], Pritam Sinharoy, PhD[2], Eric R. Gross MD, PhD[2].

[1]Department of Surgery, Leiden University Medical Center, PO Box 9600, 2300 RC Leiden, The Netherlands.

[2]Department of Anesthesiology, Perioperative and Pain Medicine, School of Medicine, Stanford University, 300 Pasteur Drive H3580, Stanford, 94305-5640 California, The United States.

Correspondence: Eric R. Gross MD, PhD, Department of Anesthesiology, Perioperative and Pain Medicine, School of Medicine, Stanford University, email: ergross@stanford.edu,

Supplemental Material:

Microarray studies**:** Microarray analysis was performed using Illumina whole-genome gene expression BeadChips. The gene array expression for the other ALDH family of enzymes on the microarray (besides ALDH2, ALDH4A1, and ALDH7A1 that were presented in the non-supplemental portion of the manuscript) are presented in Supplemental Figure 1.

qPCR primer validation: Primer design and validation for ALDH2, ALDH4A1, ALDH7A1, and ALDH1A1 are presented in Supplemental Figure 2.

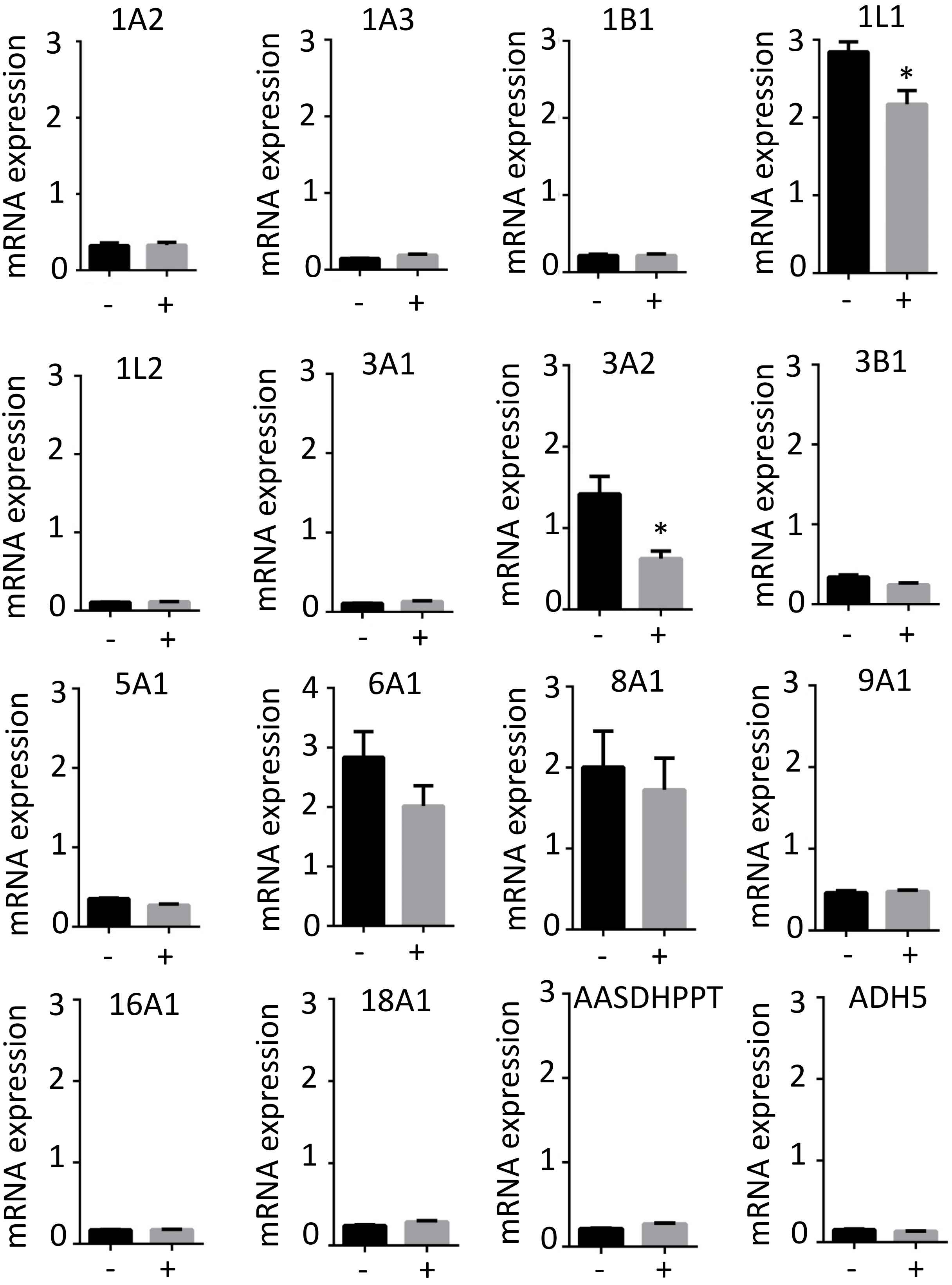

Supplemental Figure 1. ALDH family of enzymes gene array results. 16 additional ALDH enzyme family genes. Of the 16 genes, only ALDH1L1 and ALDH3A2 showed significant differences between the kidneys that did not develop delayed graft function (-) compared to those kidneys that developed delayed graft function (+). *P<0.01.

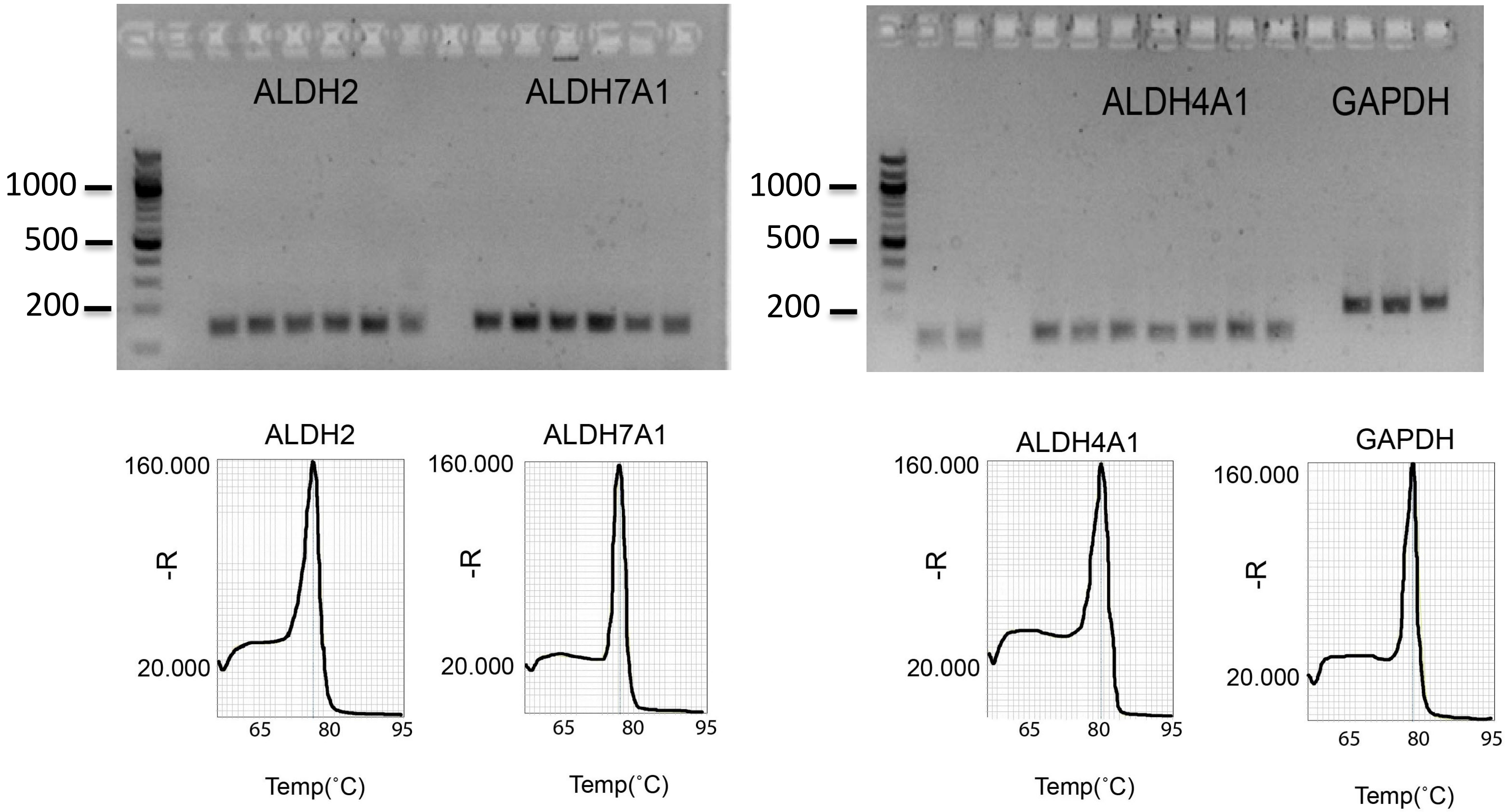

Supplemental Figure 2. qPCR studies. Gene products run on DNA gel after qPCR for each primer set used. Further representative melt curve produced for each qPCR product.
